# Stimuli Responsive Polymer-Based 3D Optical Crystals for Sensing

**DOI:** 10.3390/polym9110436

**Published:** 2017-10-25

**Authors:** Qiang Zhang, Michael J. Serpe, Samuel M. Mugo

**Affiliations:** 1 State Key Laboratory of Electroanalytical Chemistry, Changchun Institute of Applied Chemistry, Chinese Academy of Sciences, 5625 Renmin Street, Changchun 130022, China; qiang.zhang@ciac.ac.cn; 2 Department of Chemistry, University of Alberta, Edmonton, AB T6G 2G2, Canada; 3 Physical Sciences Department, MacEwan University, Edmonton, AB T5J 4S2, Canada

**Keywords:** stimuli responsive polymers, 3D optical crystals, chemosensors, polymer nanostructures

## Abstract

3D optical crystals have found their applications in sensing, actuation, optical devices, batteries, supercapacitors, etc. The 3D optical crystal devices are comprised of two main components: colloidal gels and nanoparticles. Nanoparticles self-assemble into face center cubic structures in colloidal gels. The inherent 3D optical crystal structure leads to display of structural colors on these devices following light impingement. As such, these optical properties have led to the utilization of these 3D optical crystals as self-reporting colorimetric sensors, which is the focus of this review paper. While there is extensive work done so far on these materials to exhaustively be covered in this review, we focus here in on: mechanism of color display, materials and preparation of 3D optical crystals, introduction of recent sensing examples, and combination of 3D optical crystals with molecular imprinting technology. The aim of this review is to familiarize the reader with recent developments in the area and to encourage further research in this field to overcome some of its challenges as well as to inspire creative innovations of these materials.

## 1. Introduction

3D Optical Crystals (3DOCs) are comprised of periodic spatial structural three-dimensional assembly with intermittent variation in refractive indices, which creates a photonic stop-band. Unlike pigments which yield color through selective light absorption at certain wavelengths, the 3DOCs yield structural color due to reflection of certain light wavelengths in the visible range dependent on their ordered lattice structure and therefore photonic stop-band. The interference light can be described using the well-known Bragg equation, modified for the photonic crystals, as shown in Equation (1).

*m* λ = 2 *n d* sin θ (1)

where *m* is the diffraction order, λ is the wavelength of the reflected light, *n* is the mean refractive index of the periodic structure, *d* is the lattice period of the crystalline direction of propagation of light, and θ is the angle between the incident light and diffraction crystal planes.

There are various examples of 3DOCs in nature such as butterfly’s wings, opal gemstones, beetles, etc. Inspired by natural opals, there is interest in photonic crystal materials, partly for use in optical devices, bright nonfading structural antireflection colors coatings, display materials, sensing devices, etc. [1]. Rather than use the top-down lithographic methods that are expensive and yield poor resolution, the convenient self-assembly of close packed 3D array colloid structures into templates is preferred for fashioning 3DOCs. Commonly used colloids include silica, polystyrene or polyacrylate nanoparticles. To extend the scope of structural color ranges, other inorganic colloids spheres such as ZnS and TiO_2_ nanoparticles have also been reported [1,2,3,4,5,6,7]. Demanding low polydispersity and low defect packing density, strategies for uniform deposition and packing of the colloidal structures involve self-assembly based on gravitational, electrostatic, and capillary forces; mechanical packing and physical confinement; thermally assisted colloidal assembly; and inkjet and electrohydrodynamic printing. Geometric nanoscale assembly based on mixtures of dissimilarly shaped nanoscale objects has also been reported [1,2,3,4,5,6,7].

To characterize the monodisperse colloidal nanoparticles assembly and the periodic structures, a critical index for the DOC performance, structural characterization is normally achieved using techniques such as X-ray photoelectron spectroscopy (XPS) and Nuclear magnetic resonance (NMR) spectroscopies, or dynamic light scattering (DLS), field-emission scanning electron microscopy with energy dispersive X-ray spectroscopy (FESEM-EDX), etc. [8,9,10,11].

This review paper focuses on stimuli responsive polymers based on 3DOCs (SRP-3DOCs) that are prepared using nanoparticles and stimuli responsive polymers. Nanoparticles self-assemble into crystal structures in stimuli responsive polymer matrixes. Stimuli responsive polymers (SRPs), also named “smart polymers”, could change their conformation of polymer chains in response to slight environmental changes such as pH [12], light [13], temperature [14], metal ions [15], and special molecules [16]. These conformation changes of SRPs lead to macroscopic changes in shape, solubility, and colors. If there is an analyte that can trigger changes of SRP in volume, it leads to distance changes between lattice periods (*d* in Equation (1)). According to Bragg equation, changes of d will cause wavelength changes of the reflected light, i.e., color changes. Therefore, we could deduce information of the analyte based on changes in colors and other optical information. Further, 3DOCs play an important role in telecommunications [17], information processing and storage [18], chemical sensors [19], solar cells [20], color imaging [21] and other important applications [22]. Here, we will mainly highlight its applications in sensing. 3DOCs exhibits a promising prospect as sensors due to its unique properties such as their low cost, ease of instrumental readout, simple operation, and portability. The aim of this review is to familiarize the reader with recent developments of 3DOCs in the area of sensing, encourage further research in this field to overcome some of its challenges and inspire new innovations for these materials.

## 2. Composition of Responsive Elements

### 2.1. Responsive Colloid Particles

**Figure 1:**
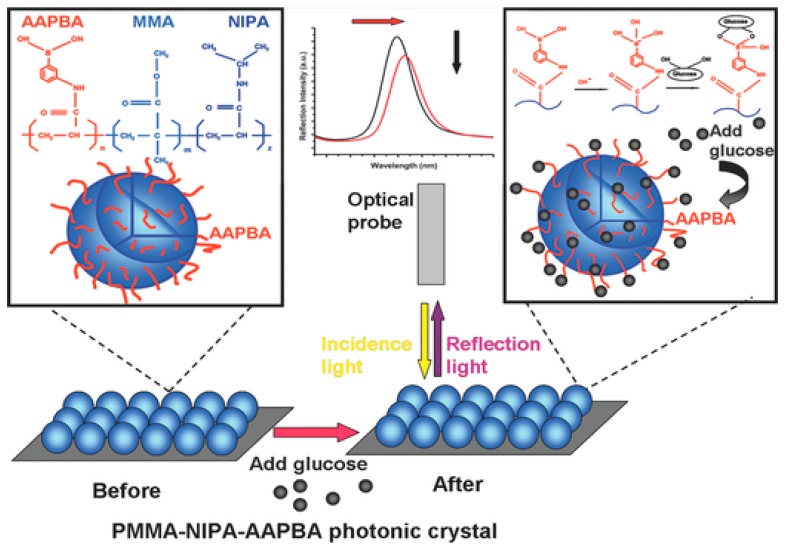
Experimental procedure for the reflectometric glucose detection using microsphere-based 3DOCs. Reproduced with permission from [26].

**Figure 2:**
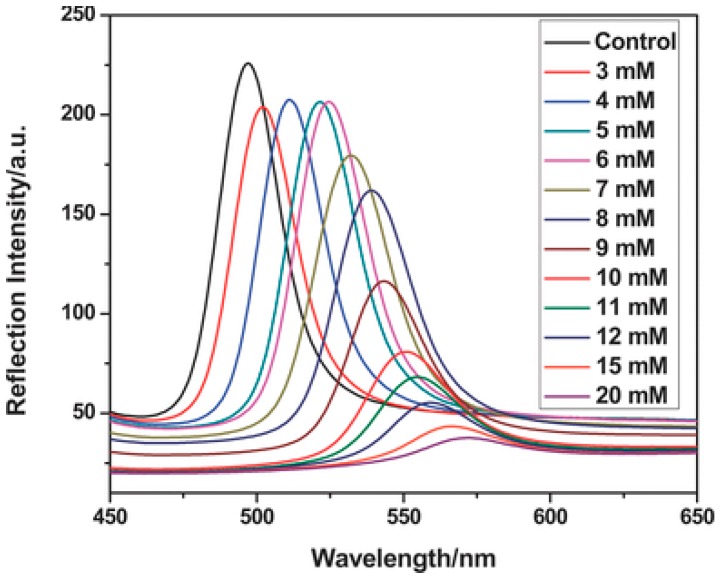
Optical responses of PNA OCP PC to different glucose concentrations. Note: PNA: MMA–NIPA–AAPBA; OCP: opal closest-packing; PC: photonic crystal. Reproduced with permission from [26].

In order to make changes in optical properties, two factors should be changed in Equation (1): refractive index or periodicity. It is improbable to allow significant changes in refractive indexes of materials. Scientists put their focus on how to change periodicity of colloid particles. Rigid nanoparticles, such as polystyrene, poly(methyl methacrylate), and silica, do not exhibit discernable swelling, deswelling, or deformation in response to environmental changes such as temperature, electric field or ionic strength. Therefore, it is improper to use these rigid nanoparticles as responsive colloid particles to prepare 3DOCs. On the other hand, hydrogel particles are utilized as responsive colloid particles in preparation of 3DOCs due to their tunable changes in sizes triggered by environmental stimuli. Poly(*N*-isopropyl acrylamide) (pNIPAm) based nanogels have hitherto attracted much attention because pNIPAm exhibits a phase transition temperature of 32 °C [23,24]. When pNIPAm is heated through 32 °C, it changes from being hydrophilic to hydrophobic. This process leads to macroscopic changes in solubility, size, and volume. In one case, thermoresponsive photonic crystals were reported by Lyon group, which were prepared using pNIPAm-based nanogels [25]. As temperature increased from 26 to 34 °C, the size of the nanogel reduced from 210 to 140 nm. The changes in size of nanogels lead to 3DOC color variation in the range of red to blue. Immobilization of recognition groups in hydrogel particles allows special detection capability of 3DOCs toward a certain analyte. For instance, phenylborate groups were incorporated into monodisperse microspheres that were synthesized by free-radical precipitation copolymerization of methyl methacrylate (MMA), NIPAm, and 3-acrylamidophenylboronic acid (AAPBA), as shown in Figure 1 [26]. The microsphere-based 3DOCs (PNA (MMA–NIPA–AAPBA) OCP (opal closest-packing) PC (photonic crystal)) were prepared and used to detect glucose because of interaction between borate groups and glucose. Glucose could bond with phenylborate groups, increasing hydrophilicity of microspheres, which makes microspheres swell. The swelling of microspheres increases lattice spaces and diameters (d). According to Bragg equation, red shift of spectra was expected with increasing glucose concentration. As expected, a red-shift wavelength of 75 nm was observed as glucose concentration increased from 3 to 20 μM (Figure 2). It should be noted that the microsphere-based 3DOCs exhibited obvious color changes (from brilliant blue to emerald green). Thus, 3DOCs exhibit a potential ability as colorimetric sensors for diabetes.

### 2.2. Responsive Polymer Matrixes

**Figure 3:**
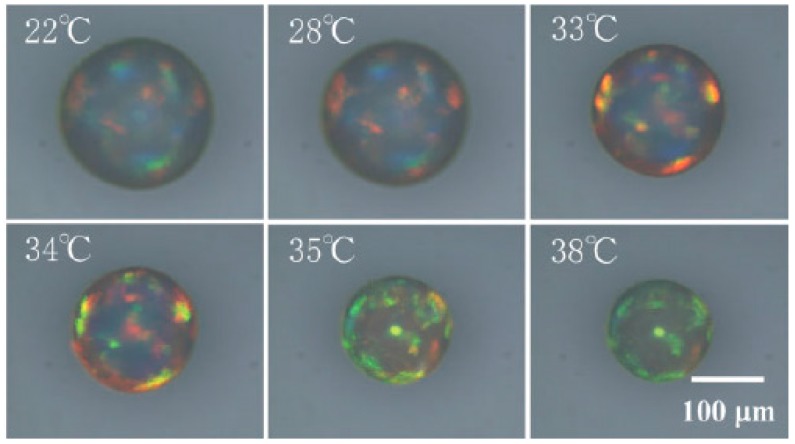
Photographs of the reflection at normal incidence of a thermosensitive PNIPAm-immobilized colloidal crystal sphere at various temperatures. Reproduced with permission from [27].

Other than responsive colloid particles, there are various other options to synthesize responsive matrixes. Responsive polymer matrixes can be synthesized by copolymerization of monomers with functional groups. Swelling or deswelling of responsive matrixes can be triggered using external stimuli, which leads to changes in lattice spaces of 3DOCs and optical spectra shifts. Kanai et al. reported a colloidal crystal system that was fabricated through combination of microfluidics and photopolymerization [27]. 3DOCs comprised of polystyrene nanoparticles (200 nm) and pNIPAm hydrogel matrixes. Due to thermal responsiveness of pNIPAm, when these colloidal crystal spheres are heated from 22 to 30 °C, the color of these spheres changed from green opal to red opal, as shown in Figure 3. This phenomenon came from shrinkage of pNIPAm hydrogels when heating through LCST [27].

## 3. Sensing Applications

### 3.1. Chemical Sensors

**Figure 4:**
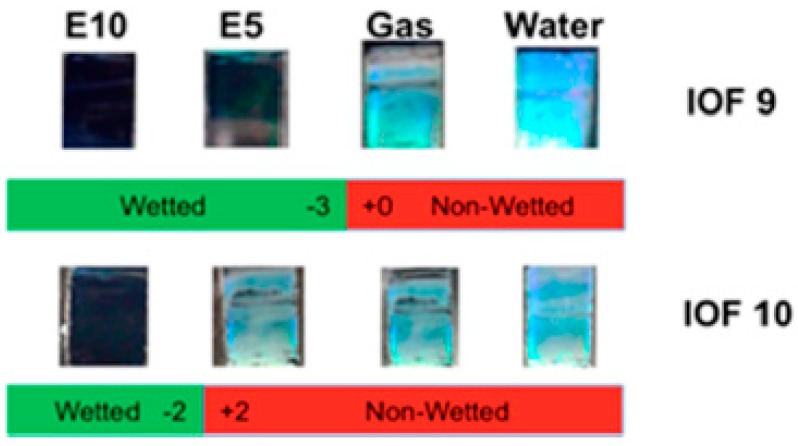
Optical images show the wettability tests on the coated IOF strips when dipped in gasoline/ethanol mixtures. Reproduced with permission from [28].

Alcohol can cause a change in refractive index or lattice spaces of 3DOCs through swelling of hydrogel matrixes. Li and coworkers developed inverse opal silica films with a three-dimensional porous structure as dip-in indicators to visually detect ethanol in gasoline [28]. The inverse opal films have a three-dimensional porous structure with a highly ordered periodic arrangement of nanopores, which exhibits a color of green due to its 3D crystal structure. When a liquid with similar refractive index with matrix enters nanopores of films, the film will lose its color (Figure 4). This phenomenon provides a powerful approach to differentiate various liquid fuel mixtures. This indicators are capable of detect 0.4% of ethanol content in the fuel mixture.

**Figure 5:**
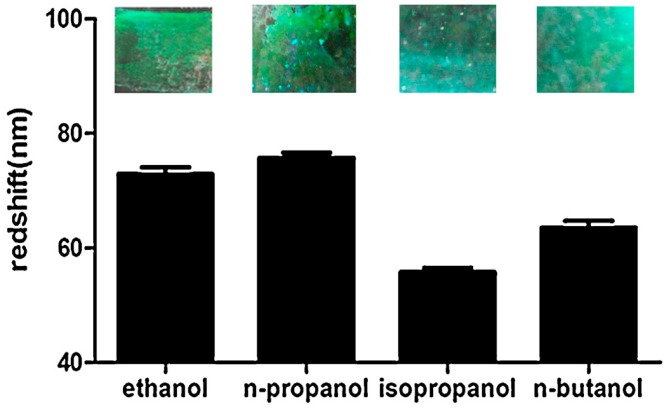
The red shift and structural color of 3DOCs in different alcohols. Reproduced with permission from [29].

Further, Wang et al., reported cellulose-based 3DOCs films used for distinguishing alcohols (ethanol, *n*-propanol, isopropanol, and *n*-butanol) [29]. The 3DOCs were prepared by infiltrating the voids of poly(methyl methacrylate) (PMMA) colloidal arrays with methyl cellulose aqueous solution, followed by thermal curing. When the film was immersed into alcohol solutions, its color changed from blue to green (Figure 5). In this process, PMMA colloidal particles in 3DOCs underwent swelling following alcohol exposure, which expanded lattice space of 3DOCs. Meanwhile, introduction of alcohols also caused increase in the average refractive index. Combination of these two factors led to a red shift of the reflection of incident light. The amplitude of red shift depends on the concentration of alcohols.

**Figure 6:**
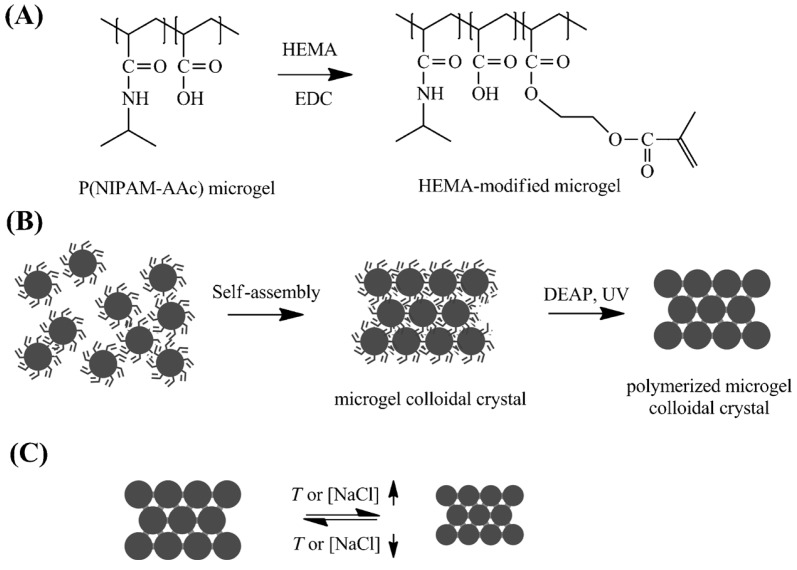
(**A**) Synthesis of HEMA-modified microgels by coupling of P(NIPAm-AAc) microgels with HEMA under catalysis by EDC. (**B**) Synthesis of polymerized microgel colloidal crystals (PMCC). (**C**) Response of PMCC to temperature or salt. Note: HEMA: 2-hydroxyethyl methacrylate; AAc: acrylic acid; EDC: *N*-(3-dimethyla-minopropyl)-*N*’-ethylcarbodiimide hydrochlorid. Reproduced with permission from [30].

**Figure 7:**
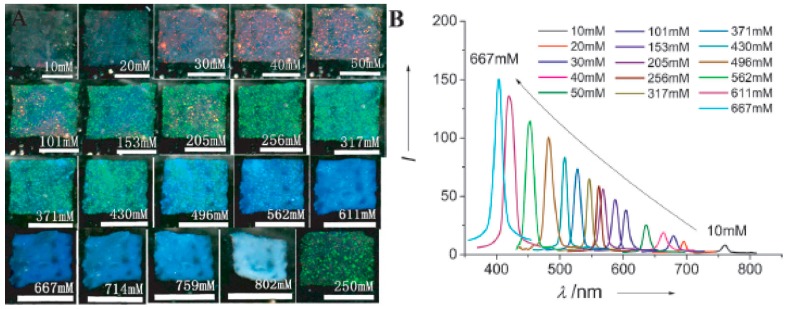
(**A**) Photographs of a freestanding PMCC film taken with [NaCl] increasing from 0 to 750 mM. The last one (bottom right) was taken when [NaCl] was decreased back to 250 mM. Scale bar: 0.5 cm. pH 3.0. *T* = 238 °C. (**B**) Reflection spectra of the PMCC film measured at various [NaCl] values. Reproduced with permission from [30].

In most cases, researchers utilize hard microspheres, such as polystyrene (PS), PMMA, and SiO_2_ particles, to fabricate 3DOCs. Compared with these hard 3DOC-based microspheres, soft 3DOC-based microspheres are intrinsically defect tolerant due to their soft nature. Chen et al. prepared photonic colloidal crystals using pNIPAm-based microgels, which exhibit tunable band gaps and fast response rates [30]. Firstly, the pNIPAm microgels with double bonds were self-assembled into colloidal crystals. Then, the crystal structure was fixed by UV mediated crosslinking these microgels, as shown in Figure 6. The resulting colloidal crystals can respond to temperature change due to the intrinsic thermal responsiveness of pNIPAm. On the other hand, ionic strength can also be used to tune the color and optical properties. In original state, the films are transparent and colorless at low NaCl concentration (<10 mM). As NaCl concentration increases to 40, 256 and 562 mM, films turn to red, green, and blue, respectively (Figure 7). This result is attributed to salt-induced deswelling of the microgel particles in the film, which leads to a smaller lattice constant of the film and an increased refractive index of the microgels.

In general, two approaches are used to fabricate 3DOCs named as in-situ self-assembly and infiltration. The first method gets involved with mixing of nanoparticles and pre-polymer solutions. The nanoparticles self-assemble into crystal structures, followed by polymerization of pre-polymer solutions forming 3DOCs. The method of infiltration contains three steps preparation process. First, nanoparticles crystallize on the surfaces of substances through evaporation process. Then, pre-polymer solutions infiltrate in the interspace of nanoparticles. Finally, polymerization of monomers is triggered using UV or initiators generating 3DOCs. Several methods have been used to extend sensing fields of 3DOCs through modifying 3DOCs with other functional components or combination of 3DOCs with other technologies. For example, molecular recognition groups were immobilized into hydrogel matrixes to detect certain analytes. In one case, a photonic crystal glucose-sensing film was prepared using polyacrylamide-poly(ethylene glycol) hydrogel with pendant phenylboronic acid groups as responsive matrix in which crystal colloidal arrays embedded [31]. The detection experiment was implemented by soaking the samples in glucose solutions for a certain time. Glucose diffuses through hydrogel matrixes because of their porous structures, followed by entering crystal colloidal particles. Glucose bonds with phenylboronic acid groups of 3DOCs through the formation of a bis-bidentate crosslink resulting in shrinkage of the hydrogel volume. These volume changes lead to the blue shift diffractions from the embedded 3DOCs that are in proportion to the glucose concentration. The color changes shift across the visible spectral region from red to blue over physiologically relevant glucose concentrations.

**Figure 8:**
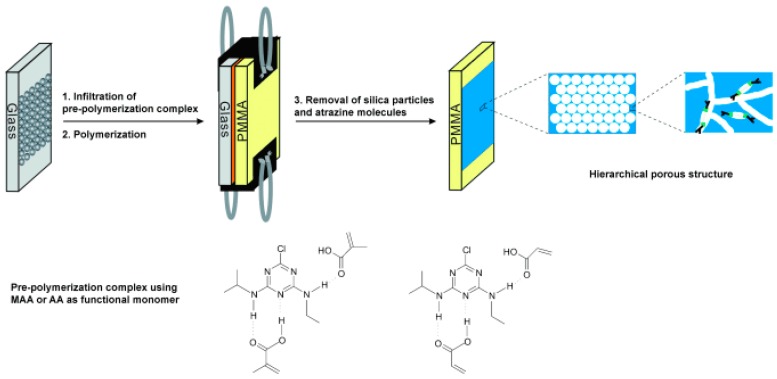
Schematic illustration of the procedure used for the preparation of the molecularly imprinted photonic polymer. Reproduced with permission from [32].

Besides incorporation of molecular recognition agents into 3DOCs, molecular imprinting is another facile method to allow 3DOCs to detect various analytes. Imprinting molecular technology creates specific molecular recognition nanocavities in polymeric matrixes of 3DOCs, which can act as artificial antibodies and exhibit high selectivity towards the imprinted molecules. For example, atrazine-imprinted 3DOCs are prepared through three steps: the preparation of a colloidal–crystal template; the polymerization of the pre-ordered complex of atrazine with functional monomers in the interspaces of the colloidal crystal; and the removal of the used templates (colloid particles and atrazine molecules) (Figure 8) [32]. Atrazine is one of the most widely used herbicides for combating weeds in farm. The atrazine nanocavities distributed in hydrogels allow 3DOCs to recognize atrazine with high specificity. The atrazine recognition process generates a readable optical signal through a change in Bragg diffraction and thereby induces color changes. Ultrasensitive (as low as 10^−8^ ng·mL^−1^) and rapid (less than 30 s) detection of atrazine in aqueous media is achieved using this sensory system.

**Figure 9:**
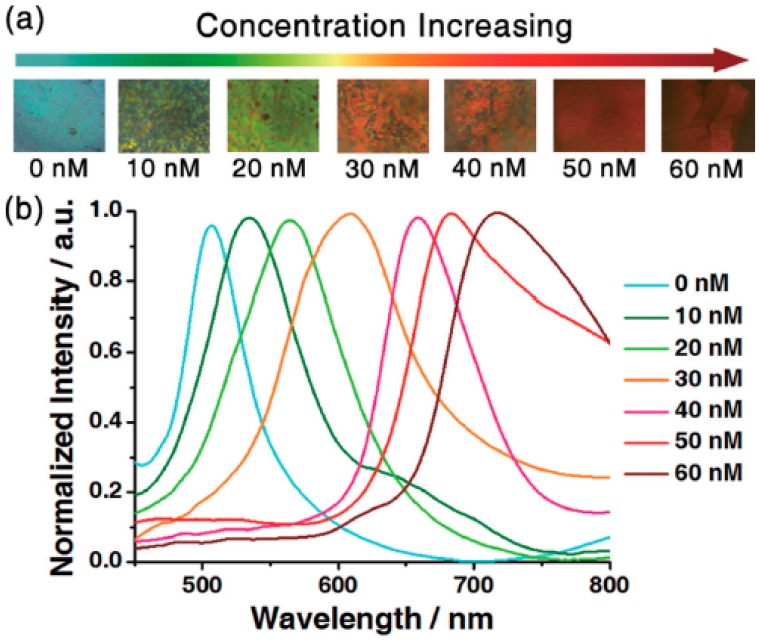
(**a**) The colorimetric transition of the molecularly imprinted polymer-photonic crystals dot as the tetracycline concentration increasing. (**b**) The spectra with different concentrations dried on MIP-PC dots with a diameter of 1.35 mm. Reproduced with permission from [33].

In another case, molecularly imprinted photonic crystal sensors were fabricated for colorimetric detection of tetracycline [33]. It was prepared by filling precursor solution in the space of opal PC dot assembled by monodispersed colloidal spheres. Subsequently, polymerization of the precursor solution was triggered with UV lamp (100 W). After polymerization, template tetracycline molecules and the colloidal spheres were removed. The hydrophilic sensors in sphere shape were prepared on the surface of hydrophobic polydimethyl sioloxane (PDMS). When a drop of tetracycline was added to the sensor, the hydrophilic-hydrophobic pattern enriches tetracycline into hydrophilic sensor’s nanopores with water evaporation. The colors of sensor dots changed from cyan to dark red as tetracycline concentration from 0 × 10^−9^ to 60 × 10^−9^ M with maximum peak shift of 208 nm, which can be recognized with naked eyes (Figure 9).

Numerous other 3D optical crystal devices have been fabricated for diverse sensing applications. Notably, Luan et al., 2017 developed silica colloidal crystal microbeads with molecular imprinting for multiplex fluorescent immunoassay [34]. Lu et al., 2017 developed a colorimetric sensor arrays for nitroaromatic compounds detection [35]. Similar 3DOCs devices have also been demonstrated for humidity sensors, and chemical separations [36,37].

### 3.2. Biosensors

**Figure 10:**
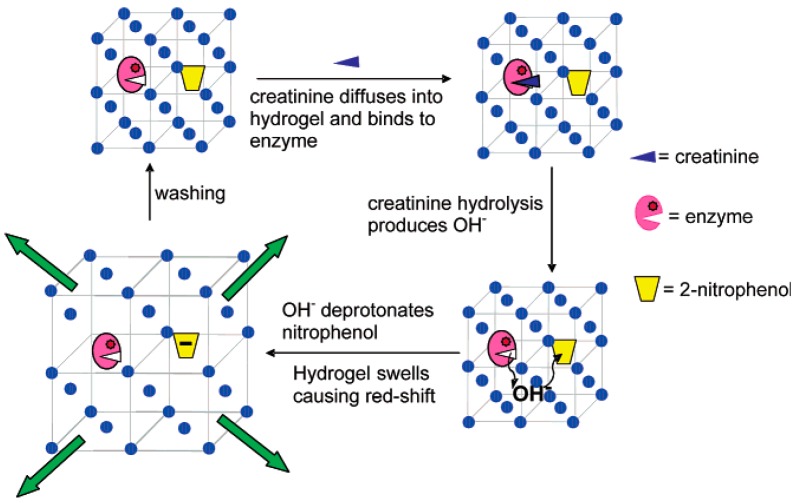
Schematic depiction of the creatinine sensor concept. Reproduced with permission from [39].

The first example of 3DOC-based biosensors was reported by Asher and coworkers who incorporated glucose oxidase (GOx) to a PCCA of polystyrene colloids [38]. Glucose caused swelling of the sensors and diffraction red shift. The swelling of sensors was attributed to the formation of a reduced flavin anion during catalyzing of glucose to gluconic acid by GOx. The oxidized and reduced flavins are uncharged and anionic at neutral pH, respectively. Flavin anion increases hydrophilicity and osmotic pressure of 3DOCs leading to the swelling of sensors. The fabricated sensors exhibited a high selectivity toward glucose over sucrose and mannose due to the inherent specificity of GOx. The Asher group further developed 3DOC-based biosensors that were used to quantitatively detect creatinine in a bodily fluid (Figure 10) [39]. Two coupled recognition agents, creatinine deiminase (CD) and 2-nitrophenol groups, were immobilized into polyacrylamide hydrogel-based matrix of 3DOCs through coupling reaction between carboxylic acid and amine groups. When creatinine was added in the system, it was hydrolyzed within hydrogels by CD releasing OH^−^. This hydrolysis reaction caused pH increasing within hydrogels, which deprotonated 2-nitrophenol groups. Compared with phenol groups, phenolate groups are more hydrophilic, which cause swelling of hydrogels and diffraction red shift. This sensor can measure creatinine at physiological levels in human blood serum.

**Figure 11:**
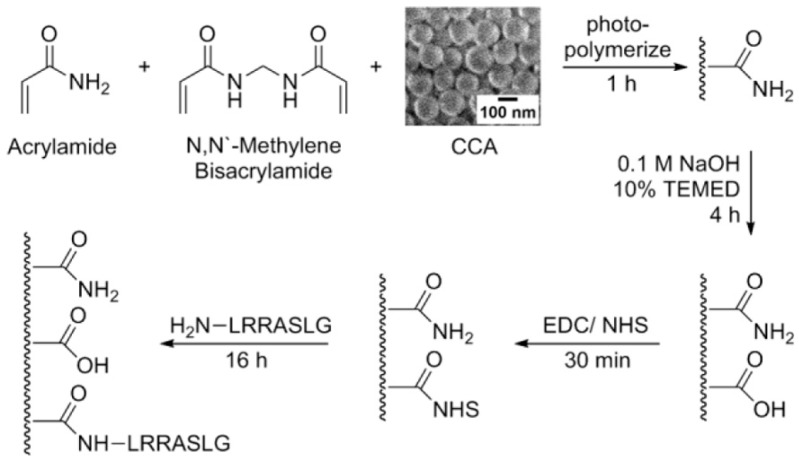
Fabrication of a Kinase Responsive CCA (crystalline colloidal array) Biosensor. Reproduced with permission from [40].

**Figure 12:**
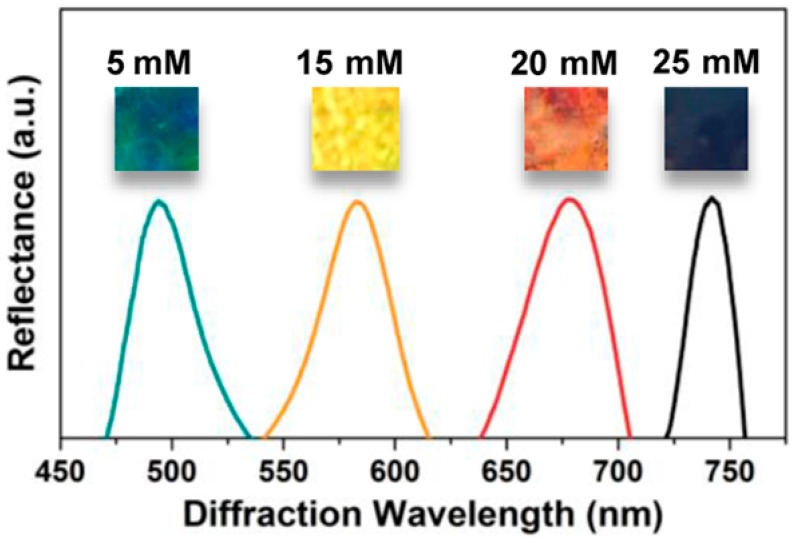
Red shift in peak optical reflectance of hydrogel-encapsulated CCAs with increasing concentration of immobilized negatively charged groups from phosphorylation of peptides (at pH 5.5). Reproduced with permission from [26].

In 2014, Stoykovich and coworkers developed a photonic crystal kinase biosensor that is based on kinase responsive hydrogels (Figure 11) [40]. Firstly, authors prepared 3DOCs through embedding polystyrene particles into polyacrylamide hydrogels. Then, polyacrylamide hydrogels were hydrolyzed with NaOH generating COOH groups. Peptides (LRRASLG or LRRApSLG) were attached to hydrogels through a coupling reaction between amine groups of peptides and COOH groups of hydrogels. The peptide sequences are responsive to kinase through phosphorylation, which results in changes in electrostatics and Donnan potential. This process leads the hydrogel to swell and the lattice spacing of the 3DOCs to increase with a concomitant red shift to the diffracted light (Figure 12).

**Figure 13:**
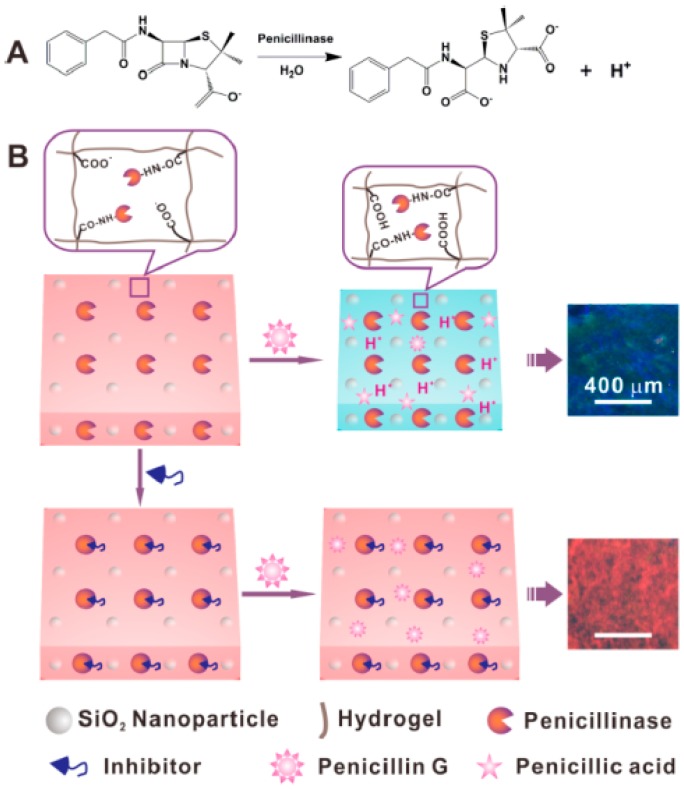
(**A**) Enzymatic reaction between penicillinase and penicillin G; (**B**) Schematic illustration of the PCCH (penicillinase colloidal crystal hydrogel) sensor for the detection of β-lactam antibiotic and β-lactamase inhibitor. Reproduced with permission from [41].

A photonic crystal was also explored to detect β-lactam antibiotic and β-lactamase inhibitor in which penicillinase (a β-lactamase) was immobilized in a pH-sensitive colloidal crystal hydrogel film [41]. When penicillin was added to the detection system, it was hydrolyzed by the penicillinase in photonic crystals producing penicillonic acid. The penicillonic acid increased local pH within photonic crystals, which causing shrinkage and blue shift diffraction in the sensors. The response process is depicted in Figure 13. The minimum detectable concentration for penicillin G was 1 μM.

### 3.3. Combination of 3DOCs with Other Technologies

**Figure 14:**
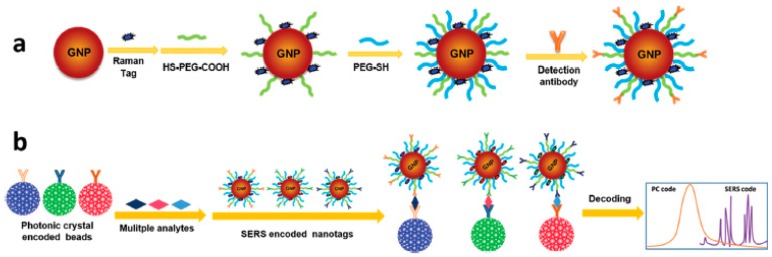
Schematic of the SERS nanotag preparation (**a**); and multiplex bioassays encoded by photonic crystal beads and SERS nanotags (**b**). Reproduced with permission from [42].

**Figure 15:**
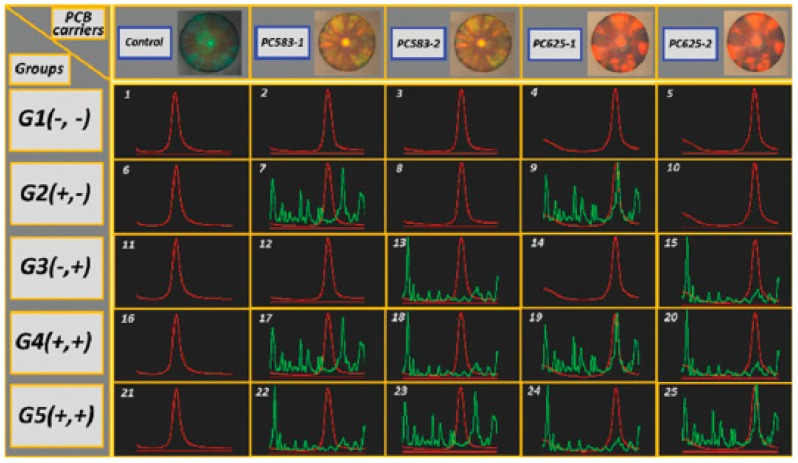
Multiplexed assays for qualitative detection of mouse IgG and rabbit IgG. In the top row are the microscopy images of three silica PCBs modified with different Fab fragments. In the middle rows are the results of multiplexed assays (red lines are reflection spectra of silica PCBs, and green lines are Raman spectra of SERS nanotags). Reproduced with permission from [42].

3DOCs as carriers and labels combined with surface enhanced Raman scattering (SERS) for the dual encoding of multiplex bioassays. Precision medicine has become one of the new generations of research in life sciences with the feature of harnessing big data. Acquisition techniques of bioinformation draw much attention to obtain both high throughput sequencing and screening based on multiplex bioassays. The combination of encoding elements and detection label in different modes make it possible to enlarge the analyte throughput by dual or complex encoding. In one case, capture antibodies were covalently attached on the surface of silica nanoparticle-based 3DOCs [28]. The silica 3DOCs were used as biomolecular carriers and an encoding element. Then, the 3DOCs were incubated in antigen solutions for 30 min to form immunocomplexes between antigens and antibodies in 3DOCs. Finally, SERS tags comprised of gold nanoparticles with Raman tags were added to the system, which yielded a sandwich structure, as shown in Figure 14. Multiplex antigens could be deciphered by the reflection peaks of photonic crystals and Raman scattering peaks of SERS nanotags (Figure 15).

**Figure 16:**
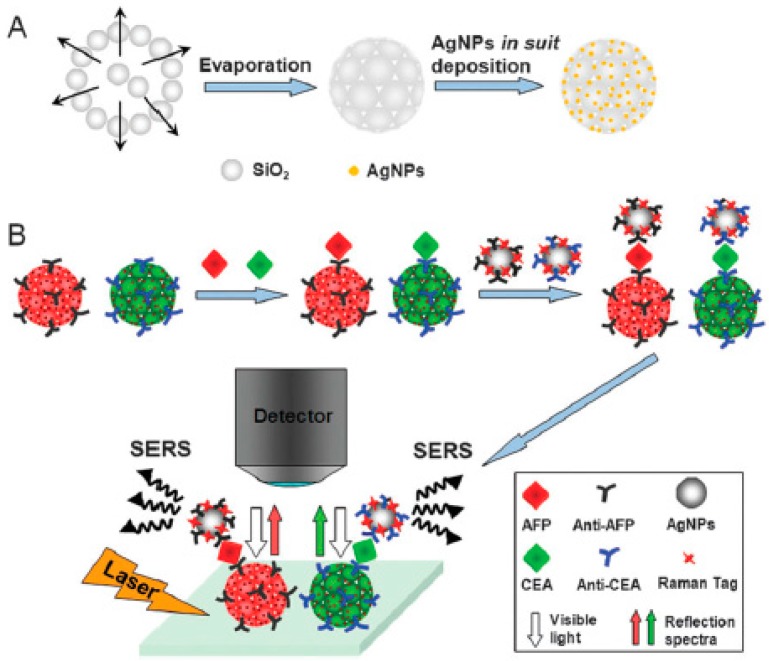
The schematic illustration of the fabrication of Ag-SPCBs (silver nanoshells silica photonic crystal beads) (**A**); and the multiplex SERS bioassay (**B**). Reproduced with permission from [43].

Another similar multiplexed assay system was prepared using silica photonic crystal beads with silver nanoshells [43]. The silica photonic crystal beads were prepared using silica nanoparticles with a diameter of 240 nm with a microfluidic device. Subsequently, silver nanoparticles were in situ deposited on the surface of silica photonic crystal beads. The antibody molecules were immobilized onto Ag-3DOCs providing bio-recognition sites for antigens. When antigens (tumor markers: carcinoembryonic antigen (CEA) and alpha-fetoprotein (AFP)) are present in the system, immunocomplexes with antibodies in Ag-3DOCs are formed. Then, silver nanoparticle-based SERS signal-amplified probes were fixed on antigens through similar bio-recognition. The preparation process was depicted in Figure 16. The sandwich structures exhibit distinct structural colors and reflection wavelengths. Based on this code information (reflectance spectra and Raman signals), multiplex SERS assay system could be used for the quantitative detection of CEA and AFP.

**Figure 17:**
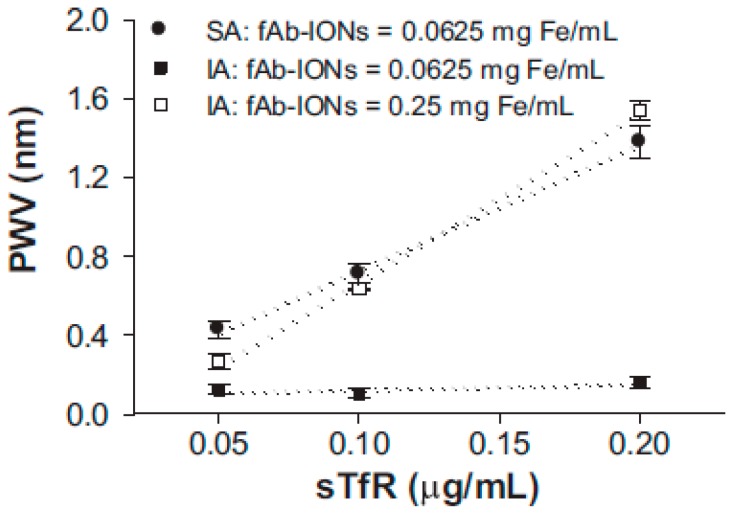
Comparison of linear responses when using inverse sandwich assay (IA) at 0.0625 and 0.25 mg Fe/mL with the standard assay (SA) at 0.0625 mg Fe/mL to measures transferrin receptor (TfR) on the 3DOCs biosensor. Iron-oxide nanoparticles with antibodies: fAb-IONs. PWV: peak shift. Responses were measured at 200 min. Reproduced with permission from [44].

Another interesting case was by Andrade and coworkers who used iron-oxide nanoparticles with antibodies to separate transferrin receptors and construct a forementioned sandwiching structure (Figure 17) [44]. When iron-oxide nanoparticles and antigens are bonded on top of photonic crystal, they will change light path resulting in diffraction shifts of the complex system [45]. The limit of detection for hemoglobin is 14 μg/mL. However, diffraction shift is very small compared with normal 3DOCs, which is below 2 nm.

While only a few examples of DOCs have been highlighted in detail herewith, there is tremendous interest and science output of design of new responsive polymeric matrices architectures with unique multi-responsivities useful for many applications, particularly drug delivery and sensing. The work of Zheng et al., 2017 [46], Halligan et al., 2017 [47], de Baubigny et al., 2017 [48], Liu et al., 2017 [49], Thonyot et al., 2017 [50], Nikjoo et al., 2017 [51] and Chacón et al., 2017 [52] are especially interesting in their design of responsive polymer 3DOCs.

## 4. Conclusions

In this review, preparation and applications of 3DOCs were illuminated with various recent examples. 3DOCs are comprised of two components: crystal nanoparticles and responsive polymeric matrixes. 3DOCs were interpreted to familiar readers with many examples based on the two components. Then, sensing applications of 3DOCs were discussed in the second part, which also covered the fabrication methods demonstrated using these examples. Besides these, as a new development direction of 3DOCs, combination of 3DOCs with other techniques was also brought up, which enlarge the analyte throughput by dual or complex encoding. Specifically, these 3DOCs were successfully used as chemical sensors and biosensors, which can be read out by observing changes in colors and diffraction shifts of optical devices. Compared with other techniques, 3DOC-based sensors exhibit many advantages, such as lower costs, easy use, and visual colorimetric detection. There are tremendous opportunities in both fundamental and applied science. In particular, most sensors were prepared based on self-assembly process, which can be carried out in general research labs without the need for expensive instruments and strict processing conditions. While much progress has been made, many challenges still exist that prevent the use of these materials in everyday applications. For example, regarding detection time, response time of 3DOC-based biosensors is generally several hours, which needs to be shortened for real applications. Regardless of the challenges, continuous development of new responsive polymers and related 3DOCs makes us optimistic about the future positive impacts these materials can have on human life.
